# Quantitative proteomics insights into *Chlamydomonas reinhardtii* thermal tolerance enhancement by a mutualistic interaction with *Sinorhizobium meliloti*

**DOI:** 10.1128/spectrum.00219-24

**Published:** 2024-07-16

**Authors:** Na Zhao, Fei Liu, Wenxiu Dong, Jie Yu, Larry J. Halverson, Bo Xie

**Affiliations:** 1 School of Life Sciences, Hubei Key Laboratory of Genetic Regulation and Integrative Biology, Central China Normal University, Wuhan, China; 2 Translational Medicine Research Center, Guizhou Medical University, Guiyang, China; 3 Department of Plant Pathology, Entomology, and Microbiology, Iowa State University, Ames, Iowa, USA; Swansea University, Swansea, United Kingdom

**Keywords:** *Chlamydomonas reinhardtii*, *Sinorhizobium meliloti*, algae-bacteria interaction, proteomics, thermal tolerance

## Abstract

Interactions between photosynthetic microalgae and bacteria impact the physiology of both partners, which influence the fitness and ecological trajectories of each partner in an environmental context-dependent manner. Thermal tolerance of *Chlamydomonas reinhardtii* can be enhanced through a mutualistic interaction with vitamin B_12_ (cobalamin)-producing *Sinorhizobium meliloti*. Here, we used label-free quantitative proteomics to reveal the metabolic networks altered by the interaction under normal and high temperatures. We created a scenario where the growth of *Sinorhizobium* requires carbon provided by *Chlamydomonas* for growth in co-cultures, and survival of *Chlamydomonas* under high temperatures relies on cobalamin and possibly other metabolites produced by *Sinorhizobium*. Differential abundance analysis identified proteins produced by each partner in co-cultures compared to mono-cultures at each temperature. Proteins involved in cobalamin production by *Sinorhizobium* increased in the presence of *Chlamydomonas* under elevated temperatures, whereas in *Chlamydomonas*, there was an increase in cobalamin-dependent methionine synthase and certain proteins associated with methylation reactions. Co-cultivation and heat stress strongly modulated the central metabolism of both partners as well as various transporters that could facilitate nutrient cross-utilization. Co-cultivation modulated expression of various components of two- or one-component signal transduction systems, transcriptional activators/regulators, or sigma factors, suggesting complex regulatory networks modulate the interaction in a temperature-dependent manner. Notably, heat and general stress-response and antioxidant proteins were upregulated in co-cultures, suggesting that the interaction is inherently stressful to each partner despite the benefits of mutualism. Our results shed insight into the metabolic tradeoffs required for mutualism and how metabolic networks are modulated by elevated temperature.

**IMPORTANCE:**

Photosynthetic microalgae are key primary producers in aquatic ecosystems, playing an important role in the global carbon cycle. Nearly every alga lives in association with a diverse community of microorganisms that influence each other and their metabolic activities or survival. One chemical produced by bacteria that influence algae is vitamin B_12_, an enzyme cofactor used for a variety of metabolic functions. The alga *Chlamydomonas reinhardtii* benefits from vitamin B_12_ produced by *Sinorhizobium meliloti* by producing the amino acid methionine under high temperatures which are required for *Chlamydomonas* thermotolerance. Yet, our understanding of this interaction under normal and stressful temperatures is poor. Here, we used quantitative proteomics to identify differentially expressed proteins to reveal metabolic adjustments made by *Chlamydomonas* and *Sinorhizobium* that could facilitate this mutualism. These findings will enhance our understanding of how photosynthetic algae and their associated microbiomes will respond as global temperatures increase.

## INTRODUCTION

Photosynthetic microalgae are key primary producers in aquatic ecosystem and play an important role in the global carbon cycle and oxygen fluxes (1). Phytoplankton are enmeshed in an organic nutrient-rich layer called the phycosphere, which is replete with various bacteria (2, 3). Extensive studies have shown that bacterial interactions with algae can greatly influence the growth of both partners, phycosphere microbiome composition, aquatic food webs, and nutrient cycles (4–8).

Vitamin B_12_ (cobalamin) is a required cofactor for more than 20 enzymes involved in a variety of metabolic pathways, for example, C1 metabolism and methionine biosynthesis, and may also be involved in gene expression regulation via B_12_-riboswitches or B_12_-dependent photoreceptors (9). The importance of B_12_ cross-feeding is great in alga-bacteria interactions, as indicated by an estimate that about 50% of eukaryotic phytoplankton species require exogenous B_12_ for growth (10, 11). This is due, in part, to the presence of a B_12_-dependent methionine synthase (METH) and the absence of a B_12_-independent methionine synthase (METE) in many algal species (12); some algae have both METE and METH. Vitamin B_12_-dependent organisms scavenge it from the environment and can increase exposure to cobalamin if they are associated with B_12_-producing bacteria that reside in the phycosphere (13–16). Recent reports also indicated that pseudocobalamin can be remolded to bioactive cobalamin by algae or bacteria, suggesting a more complex exchange of cobalamin and derivatives may exist within phycosphere-associated microbial communities (14, 17).

*Chlamydomonas reinhardtii* is a unicellular microalga found in soil and aquatic environments and has been widely used as a model in photosynthesis and ciliary/flagellar research (18). Interestingly, *C. reinhardtii* has both *METE* and *METH* genes, which likely contributes to ecological flexibility in adapting to B_12_-limited or -rich environments (10, 12). A mutualism with a B_12_-producing bacterium and an alga was shown with an experimentally evolved *C. reinhardtii* METE-deficient mutant, resulting in a B_12_-dependent derivative (19–21). When *C. reinhardtii* is exposed to a prolonged temperature upshift (for example, >40°C for multiple days), it will become chlorotic and ultimately die. However, in the presence of external B_12_- or B_12_-producing bacteria, *C. reinhardtii* thermal tolerance can be greatly enhanced (22). Central to this is that heat stress represses *C. reinhardtii METE* gene expression and exogenous B_12_ amendments can maintain METH-mediated methionine biosynthesis at high temperatures (22), indicating that B_12_ is not only important for the growth of some microalgae but can also contribute to thermal tolerance. Methionine is not only an important amino acid for protein synthesis but also a direct precursor for the major cellular methyl donor, S-adenosylmethionine (SAM). Methylation reactions for heat stress responses have been reported in various organisms previously (23), implying that B_12_-induced thermal tolerance may be associated with cellular methylation reactions. However, our understanding of the molecular details in algae and B_12_-producing bacteria interactions is still limited. A major challenge is identifying the molecular mechanisms that enable the bacterial-algal association, the specific role of B_12_ cross-feeding in establishing and maintaining the association, and how this interaction influences the fitness of each partner in a changing environment.

Recently, genome-scale (transcriptomic, metabolomic, and proteomic) approaches have been applied to dissect and identify key traits that are integral to establishing or maintaining different phytoplankton-bacteria cocultures (16, 24). For example, a proteomics study using the co-culture of B_12_-dependent alga *Lobomonas rostrate* and B_12_-producing bacterium *Mesorhizobium loti* revealed extensive changes in the algal proteome in the co-culture compared to the axenic culture, suggesting that the interaction is not restricted to the exchange of metabolites but also may involve other signaling pathways (16). In this study, we used label-free quantitative proteomics to reveal changes in the proteomes of *C. reinhardtii* 21gr (Cr 21gr) and the B_12_-producing bacterium *Sinorhizobium meliloti* 1021 (Sm 1021) in mono- and co-cultures under normal and thermal stress conditions. Here, we created a scenario where the growth of *Sinorhizobium* relies on carbon provided by *Chlamydomonas* for growth in co-cultures and the survival of *Chlamydomonas* under high temperatures relies on B_12_ and possibly other metabolites produced by *Sinorhizobium*. The differentially expressed proteins (DEPs) and the related pathways in the co-cultures under different temperatures were analyzed to predict the metabolic adjustments, including stress responses, made by *Chlamydomonas* and *Sinorhizobium* to facilitate this mutualism. These data shed new insights into the molecular bases of the mutualistic interactions between a photosynthetic microalga and B_12_-producing bacterial partner and how those cellular responses may facilitate algal thermal tolerance enhancement.

## RESULTS

**Fig 1:**
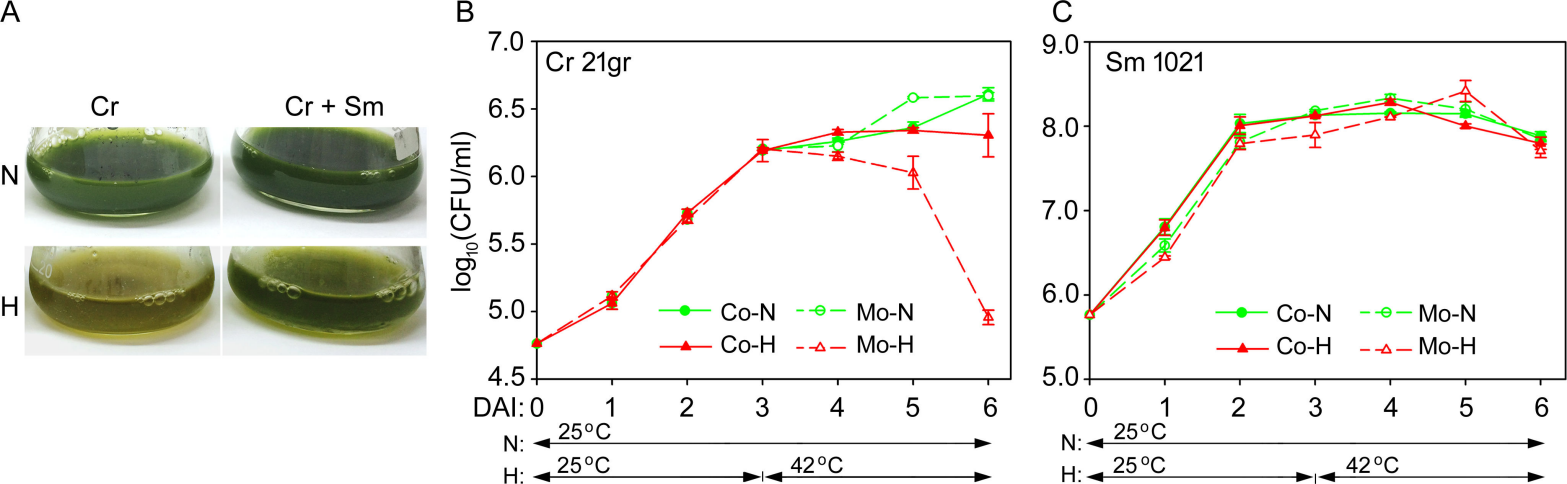
Enhancement of *C. reinhardtii* thermal tolerance by co-cultivation with *S. meliloti*. (**A**) Photographs illustrate how co-cultivation with *S. meliloti* (Sm) reduces the extent of *C. reinhardtii* (Cr) chlorosis 2 days after an upshift from normal (25°C) to high (42°C; **H**) temperatures. The controls are cultures at a normal temperature of 25°C (**N**). For the high-temperature treatment, cultures were grown at 25°C for 3 days prior to the 42°C temperature upshift. (**B and C**) Survival of *Chlamydomonas* (**B**) and *Sinorhizobium* (**C**) in different treatments. Co-H, co-culture at high temperature; Co-N, co-culture at normal temperature; Mo-H, mono-culture at high temperature; Mo-N, mono-culture at normal temperature; DAI, days after inoculation. The bottom arrows indicate the growth periods with normal temperature (25°C; **N**) or with a 42°C temperature upshift (**H**). Values are the mean ± SEM of three to four independent experiments.

Mono-cultures of *Chlamydomonas* and *Sinorhizobium* and their co-cultures were grown at 25°C (normal temperature) for 3 days prior to exposing them to a temperature upshift to 42°C (high temperature). Consistent with our previous study (22) on solid media, liquid cultures grew well at 25°C and *Chlamydomonas* mono-cultures exhibited visible chlorosis within 3 days after a 42°C temperature upshift (Fig. 1A). Following the temperature upshift, the *Chlamydomonas* population size neither increases nor decreases in the co-cultures, although it begins to decrease within 2 days after the temperature upshift in the *Chlamydomonas* monocultures (Fig. 1C). In contrast, *Sinorhizobium* survival was little influenced by co-cultivation with *Chlamydomonas* or by the temperature upshift (Fig. 1B). To reveal insight into the adaptive responses of each member of the co-culture to a temperature upshift and how the interactions between *Chlamydomonas* and *Sinorhizobium* (mono- vs co-culture) influences protein expression, we chose to examine the *Chlamydomonas* and *Sinorhizobium* proteomes 2 days after the temperature upshift since it preceded the die-off that occurred in *Chlamydomonas* monocultures.

### Overview of *C. reinhardtii* and *S. meliloti proteome*

The proteomes of *Chlamydomonas* and *Sinorhizobium* were obtained and analyzed as outlined in the “Materials and methods.” After LC-MS/MS ( liquid chromatography−tandem mass spectrometry) analysis, a total of 2,447 *C*. *reinhardtii* (about 13% of the total predicted) and 2,396 *S*. *meliloti* (about 34% of the total predicted) measurable proteins were identified. There was high congruence between experimental replicates, with correlations (*R*^2^) ranging from 0.90 to 0.97 for *C. reinhardtii* samples and 0.97 to 0.99 for *S. meliloti* samples.

**Fig 2:**
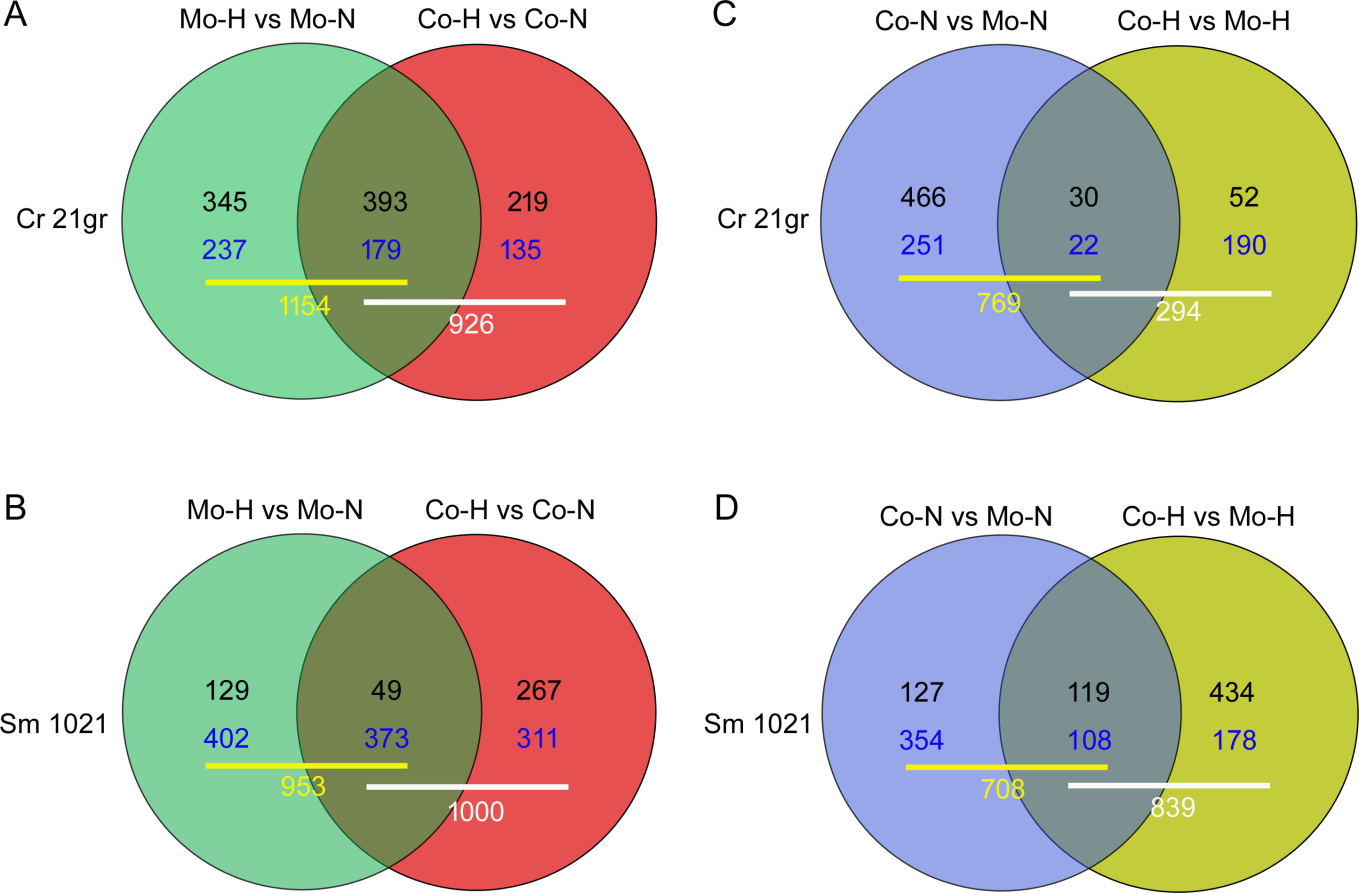
Influence of co-culture and heat stress on the number of DEPs in *C. reinhardtii* 21gr (A and C) and *S. meliloti* 1021 (B and D). (A and B) Comparisons between temperature upshift and normal temperature in mono- and co-culture. (C and D) Comparisons between co-cultures and mono-cultures under normal and temperature upshift conditions. Co-H, co-cultures following a high (42°C) temperature upshift; Co-N, co-cultures at normal temperature (25°C); Mo-H, mono-cultures at high temperature; Mo-N, mono-cultures at normal temperature. Black values are number of proteins whose expression is upregulated, and blue values are number of proteins whose expression is downregulated. White and yellow bold values are total number of DEPs.

We used distributed normalized spectral abundance factor (dNSAF), a label-free quantitative measure of protein abundance (25) based on spectral counts corrected for peptides shared by multiple proteins, for comparative proteomic analyses. Based on pair-wise comparisons, we identified DEPs in both *Chlamydomonas* and *Sinorhizobium* under each growth condition (Fig. 2; Tables S1 to S8). When comparing high vs normal temperatures, we detected slightly more differentially expressed *Chlamydomonas* proteins in the mono- than co-cultures (Fig. 2A; Tables S1 and S2), while for *Sinorhizobium*, there were no such differences (Fig. 2B; Tables S5 and S6). Interestingly, when comparing co- with mono-cultures, we detected twofold more differentially expressed *Chlamydomonas* proteins under normal compared to high-temperature upshifts (Fig. 2C; Tables S3 and S4). Yet, for *Sinorhizobium*, there were slightly more DEPs under high compared to normal temperature treatments (Fig. 2D; Tables S7 and S8). We also identified differentially expressed proteins that responded to both treatments, as illustrated by the overlapping circles in the Venn diagrams (Fig. 2). Significantly, we identified uniquely differentially expressed *Chlamydomonas* and *Sinorhizobium* proteins in response to the temperature upshift in mono- and in co-cultures, suggesting that responses to a temperature upshift can be modulated by the presence of a mutualistic partner.

### Comparative proteomics reveal changes in *Chlamydomonas* and *Sinorhizobium* metabolism at high growth temperature

**Fig 3:**
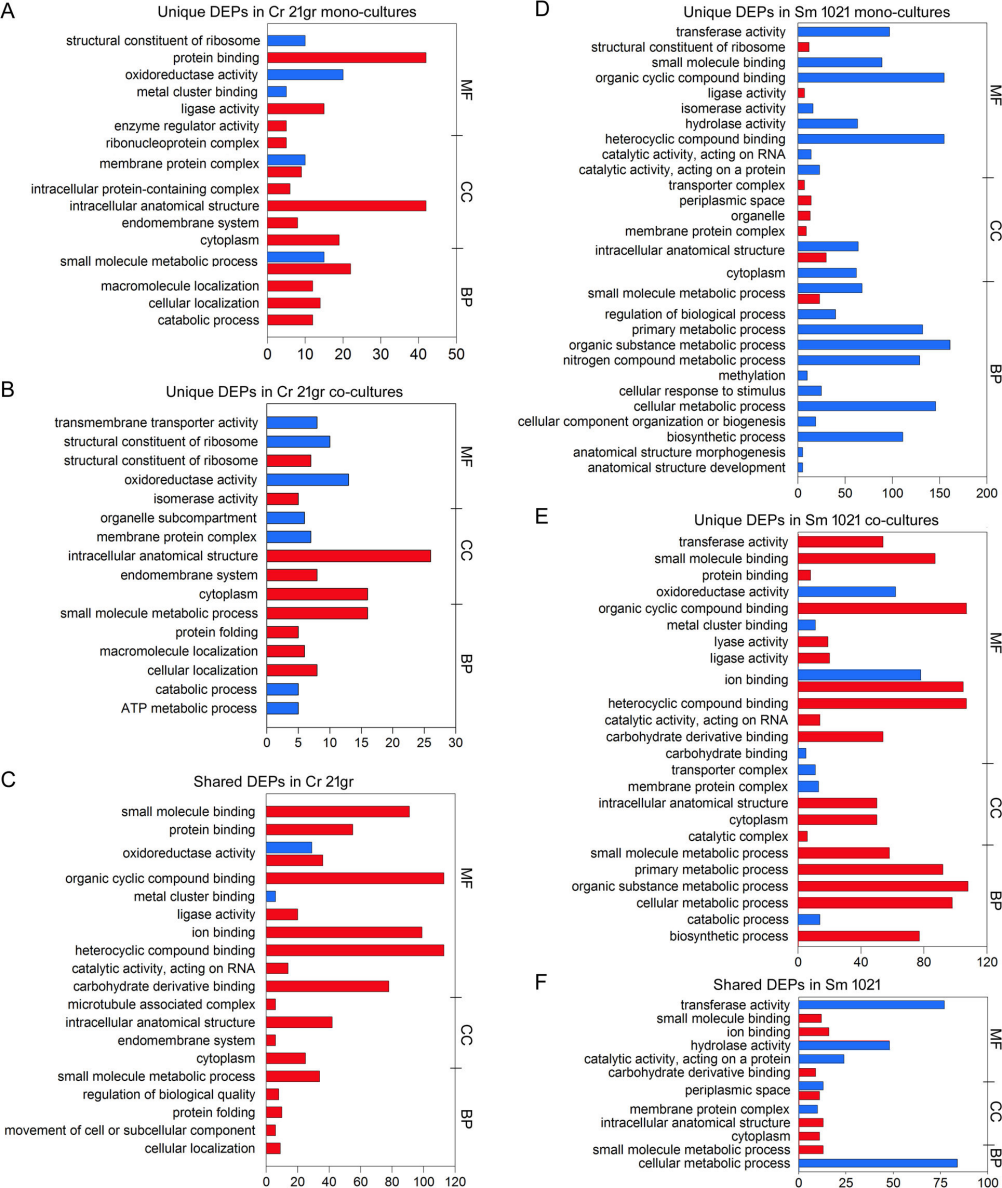
GO classifications of the upregulated (red) and downregulated (blue) DEPs *in C. reinhardtii* (A–C) and *S. meliloti* (D–F) when comparing high and normal temperature treatments. The DEPs are categorized by whether the proteins are defined to be involved in metabolic functions (MFs), biological processes (BPs), or as a cellular component (CC), as indicated on the right y-axis. (A and D) Unique DEPs in Cr 21gr or Sm 1021 mono-cultures. (B and E) Unique DEPs in Cr 21gr or Sm 1021 co-cultures. (C and F) DEPs that respond to a temperature upshift in mono- and co-cultures.

The broader gene ontology (GO) analyses revealed the magnitude and nature of how a temperature upshift in co- and mono-cultures influenced proteomes. Changes in the *Chlamydomonas* proteome when co-cultured with *Sinorhizobium* were substantial and distinct from the proteome in the absence of *Sinorhizobium*, particularly proteins associated with protein folding, structural constituents of ribosomes, and organelle subcompartments (Fig. 3A and B; Tables S1 and S2). Following heat stress, those 572 DEPs (Fig. 2A) of the *Chlamydomonas* proteome were unaffected by co-cultivation with *Sinorhizobium*, particularly small molecule binding, protein binding, organic cyclic compound binding, and heterocyclic compound binding proteins (Fig. 3C). Yet, several heat shock proteins and chaperones (e.g., CLPB4 and HSP70C) are enriched in both *Chlamydomonas* mono- and co-cultures at elevated temperatures, indicating thermal tolerance relies on the same cellular responses to heat stress in the absence or presence of *Sinorhizobium*.

The temperature-upshift had a more dramatic effect on the metabolism of *Sinorhizobium* than *Chlamydomonas* (compare Fig. 2A with 2B), and the metabolic processes most affected were those involved in organic cyclic and heterocyclic compound binding and organic substance (Fig. 3D through F). Many of the metabolic processes up-regulated in *Sinorhizobium* by a temperature upshift included those involved in small molecule metabolic process and intracellular anatomical structures. In comparison, there were more unique downregulated proteins in *Sinorhizobium* when exposed to heat stress as a mono- than as a co-culture with *Chlamydomonas* (402 compared to 311 as shown in Fig. 2B, compare Fig. 3D with 3E). When co-cultured with *Chlamydomonas* under heat stress, *Sinorhizobium* metabolism changes included, for example, the upregulation of ion binding, small molecule binding, organic substance metabolic process, and biosynthetic processes.

### Comparative proteomics reveals changes in *Chlamydomonas* and *Sinorhizobium* metabolism when grown in coculture with each other

**Fig 4:**
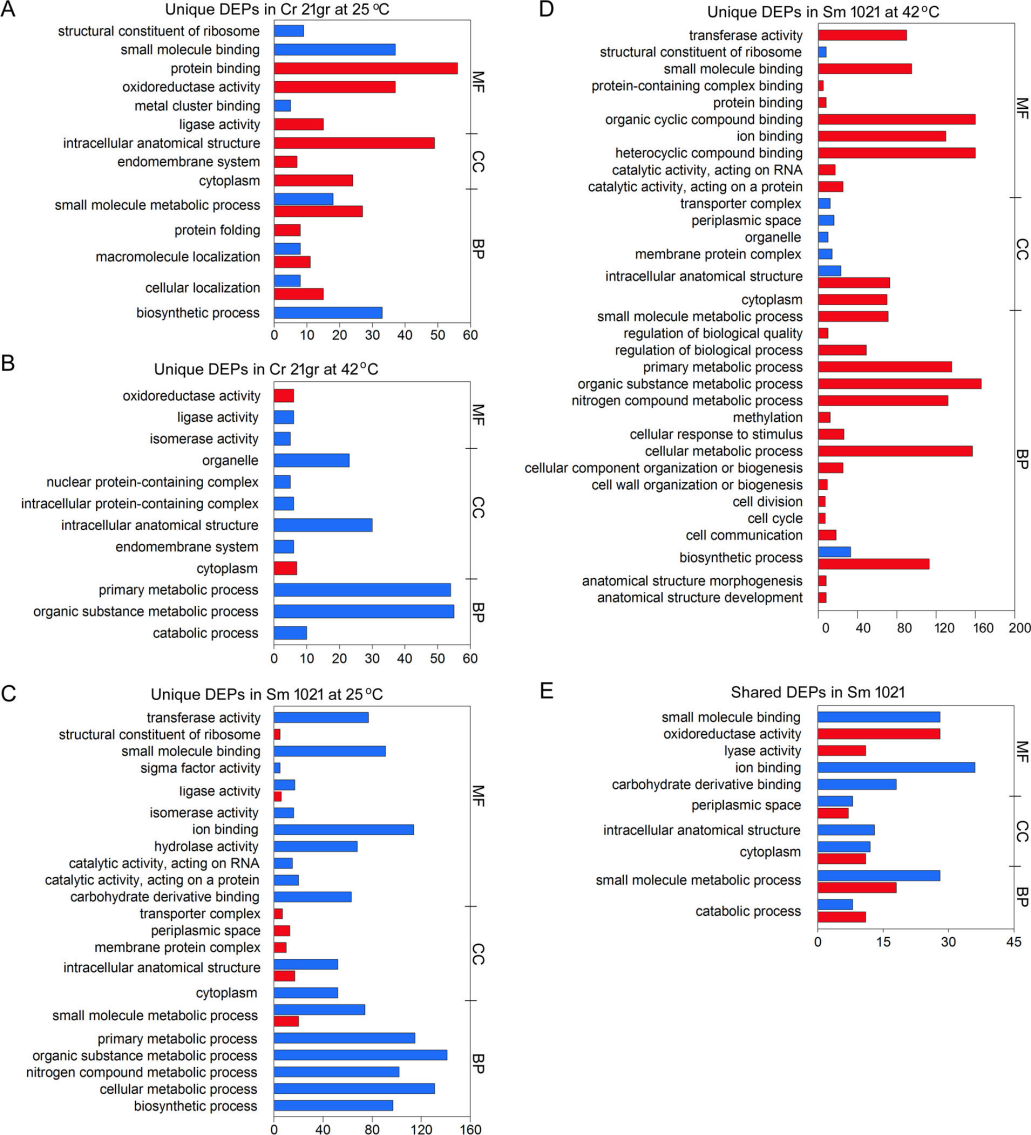
GO classifications of upregulated (red) and downregulated (blue) DEPs in *C. reinhardtii* (**A and B**) and *S. meliloti* (**C–E**) when comparing co-cultures and mono-cultures under normal (25°C) or high (42°C) temperatures. Note the absence of significant DEPs that were shared between Cr21gr mono- and co-cultures at different temperatures. The DEPs are categorized by whether the proteins are defined to be involved in metabolic functions (MFs), cellular component (CC), or biological processes (BPs). (**A and C**) Unique DEPs in Cr 21gr or Sm 1021 co-cultures at 25°C. (**B and D**) Unique DEPs in Cr 21gr or Sm 1021 co-cultures after a 42°C temperature upshift. (E) DEPs that are shared in Sm 1021 mono- and in co-cultures.

The GO analyses also revealed that *Chlamydomonas* and *Sinorhizobium* proteomes were strongly influenced by interactions with each other, although the extent of the response was modulated by heat stress (Fig. 4). Many processes, such as organic substance metabolic process, primary metabolic process, and intracellular anatomical structure, were downregulated in *Chlamydomonas* co-cultures under heat stress (Fig. 4B). Following the temperature upshift, however, the magnitude of metabolic changes was vastly greater in *Sinorhizobium* than in *Chlamydomonas* co-cultures, with an expression of the majority of proteins upregulated (28 compared to 2, compare Fig. 4D and B). The processes most affected by heat-stress in *Sinorhizobium* co-cultures were involved in organic cyclic and heterocyclic compound binding and various metabolic (organic substance, nitrogen, biosynthetic, cellular, and primary) processes (Fig. 4D). Importantly, there were few proteins in *Chlamydomonas* (such as DNJ8 and DAD1) and *Sinorhizobium* (such as Sma1231 and AatB) co-cultures whose expression was not influenced by growth temperature (Fig. 2C and D), which may be essential or contribute to maintaining the mutualistic association.

### Heat stress and co-culturing with *S. meliloti* alter *C. reinhardtii* methionine biosynthesis

**Fig 5:**
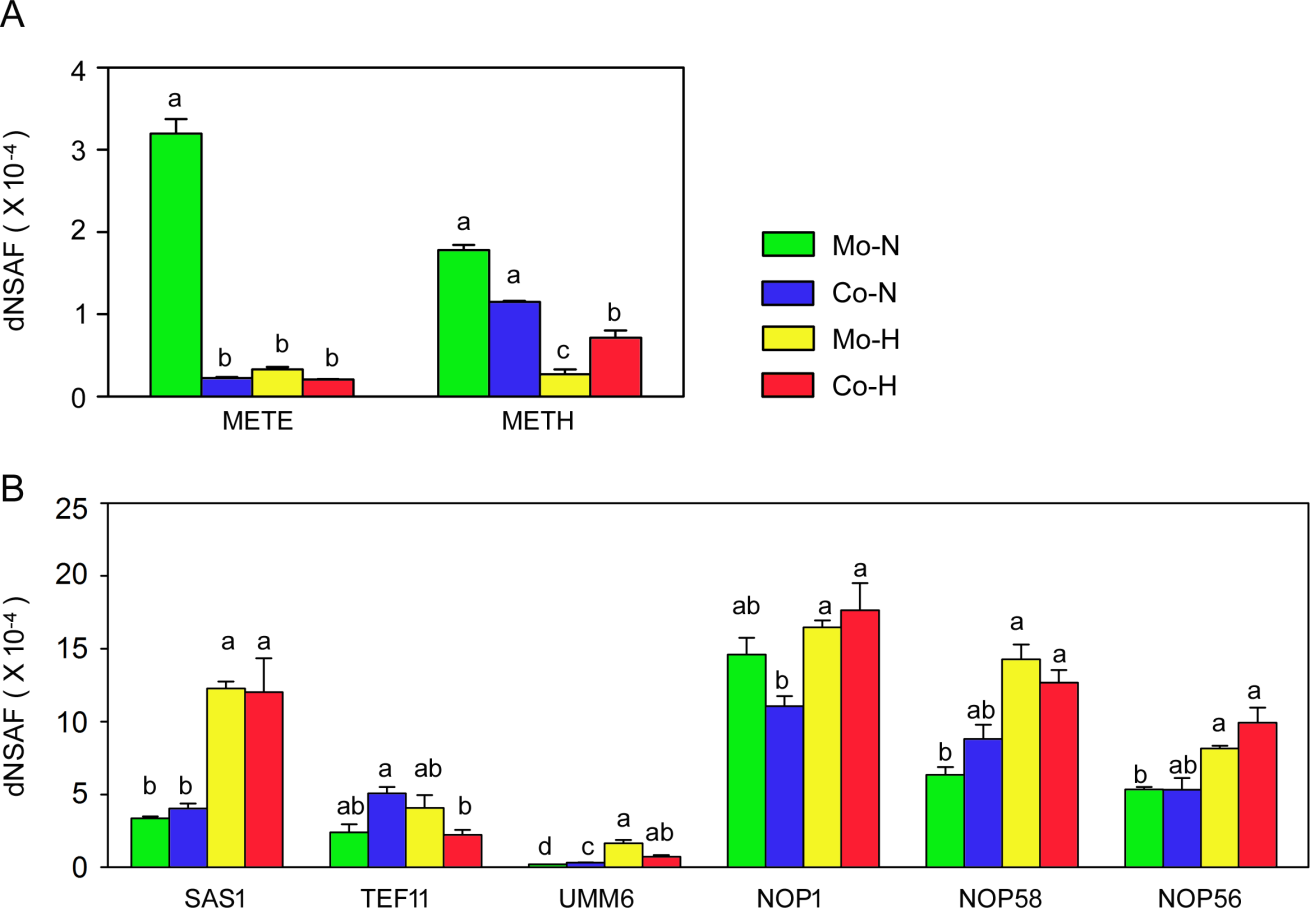
Expression levels of select proteins involved in *C. reinhardtii* methionine synthesis and methylation. (**A**) Abundance of cobalamin-independent methionine synthase METE and cobalamin-dependent methionine synthase METH. (**B**) Abundance of selected proteins involved S-adenosylmethionine synthesis and methylation reaction. SAS1, S-adenosylmethionine synthetase; TEF11, S-isoprenylcysteine O-methyltransferase related protein; UMM6, UbiE/COQ5 methyltransferase family protein. NOP1, NOP58, and NOP56, rRNA methyltransferase family proteins. Values represent the mean ± SEM of 3–4 independent experiments, and for each protein, bars marked with the same letter are not significantly different based on a one-way ANOVA with a Duncan post-hoc test (*P* < 0.05). Mo-N, mono-cultures at normal temperature (25°C); Co-N, co-cultures at normal temperature; Mo-H, mono-cultures at high temperature (42°C) upshift; Co-H, co-cultures following a high-temperature upshift.

Consistent with a previous report (22), *Chlamydomonas* under normal temperature treatments relies on the cobalamin-independent METE (Cre03.g180750.t1.2) rather than the cobalamin-dependent METH (Cre06.g250902.t1.1) methionine synthase (Fig. 5A). Moreover, in co-culture following a temperature upshift, there was nearly a 2.5-fold increase in cobalamin-dependent methionine synthase METH expression (Fig. 5A) compared to *Chlamydomonas* monocultures. Given that methionine contributes to cellular SAM production, it is not surprising that we observed increased expression of SAM synthesis and SAM-dependent methylation reactions in *Chlamydomonas* following a temperature up-shift (Fig. 5B).

### Co-cultivation with *C. reinhardtii* and high temperature increases expression of *S. meliloti* proteins for B_12_ biosynthesis

**Fig 6:**
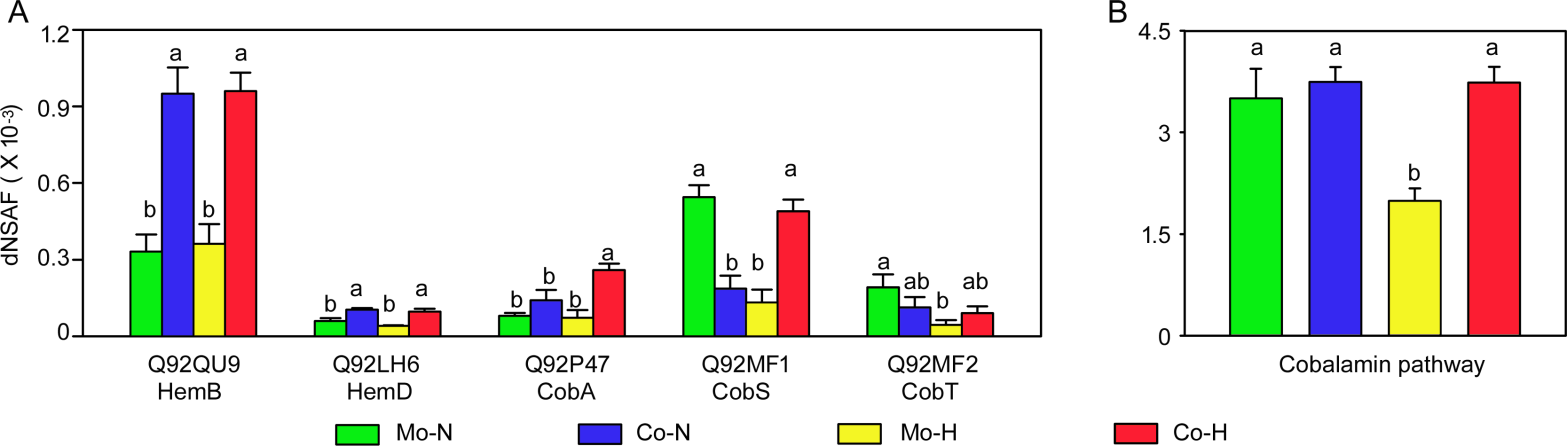
Expression levels of proteins involved in *S. meliloti* B_12_ synthesis. (**A**) Representive proteins involved in B_12_ synthesis. (**B**) Sum of dNSAF values of B_12_ synthesis pathway proteins. Values represent the mean ± SEM of four independent experiments, and for each protein, the bars marked with the same letter are not significantly different based on a one-way ANOVA with a Duncan post-hoc test (*P* < 0.05). Mo-N, mono-cultures at normal temperature (25°C); Co-N, co-cultures at normal temperature; Mo-H, mono-cultures at high temperature (42°C) upshift; Co-H, co-cultures following a high-temperature upshift.

*De novo* cobalamin biosynthesis is complex and includes the production of the tetrapyrrole uroporphyrinogenIII which is converted to precorrin-2, an intermediate in the aerobic pathway of cobalamin synthesis by *Sinorhizobium* (26). In *Sinorhizobium* co-cultures, the expression of several cobalamin and uroporphyrinogenIII biosynthesis-related proteins was upregulated (Fig. 6A; Table S9). Based on summing the dNSAF values of these proteins involved in cobalamin biosynthesis, there is a significant (*P* = 0.003) increase in B_12_ biosynthesis pathway expression by *Sinorhizobium* in co-cultures under high-temperature condition (Fig. 6B). Interestingly, we observed significantly increased expression of components of a high-affinity B_12_ uptake system (*btuBCDF*) salvage pathway in *Sinorhizobium* during co-culture with *Chlamydomonas* (Table 1) under normal and high temperatures, although at levels that were slightly below our 1.5-fold differential expression threshold.

**TABLE 1:** Expression of representative transporters^*a*^

			Fold change			
Protein ID	Gene	Functional description	H:N^*b*^		Co-:Mono-culture	
			Mono-culture	Co-culture	N	H
*C. reinhardtii* 21gr						
Cre16.g663600.t1.2	*PHT7*	Sodium-dependent phosphate transporter	1.26	**3.01**	0.78	**1.85**
Cre03.g168550.t1.1	*AOT3*	Amino acid transporter	**0.44**	1.51	0.96	**3.33**
Cre06.g289150.t1.1	*MTP5*	Cation efflux transporter, membrane protein	0.9	**0.59**	**1.86**	1.22
Cre06.g257000.t1.2	*SULP3*	Component of chloroplast transporter SulP	**3.94**	**2.17**	**2.34**	1.29
Cre15.g637761.t1.2		ABC transporter domain-containing protein	**2.71**	0.56	**4.27**	0.88
Cre10.g443000.t1.2	*ATM3*	Half-size ABC transporter	**2.99**	0.93	**1.55**	**0.48**
*S. meliloti* 1021						
Q92SA1	*pstB*	Phosphate import ATP-binding protein PstB (ABC phosphate transporter)	**0.22**	1.72	0.7	**5.49**
Q92LL5	*sitA*	Manganese ABC transporter periplasmic substrate-binding protein	**0.44**	**1.34**	0.92	**2.83**
Q92MC6	*hisX*	Histidine ABC transporter, periplasmic solute-binding protein	1.38	0.89	**2.71**	1.72
Q92W78	*sm_b20485*	Putative sugar ABC transporter ATP-binding protein	**0.34**	1.17	0.71	**2.45**
Q92TD7	*smc02774*	Putative D- or L-fucose, or pyruvic acid, periplasmic substrate-binding component of ABC transporter	0.92	**0.66**	**3.1**	**2.22**
Q92RV7	*dppD1*	Putative amino acid or peptide ABC transporter	1.00	0.65	0.48	**0.31**
Q92WK7	*sm_b20346*	Putative efflux transmembrane protein	0.81	**2.84**	**2.09**	**7.33**
Q92LW4	*smc03168*	Multidrug resistance efflux system	0.81	2.46	**2.51**	**7.64**
Q92XA5	*btuC*	ABC transporter, B_12_ transportation	**0.53**	**0.53**	**1.36**	**1.37**
Q92XA6	*btuF*	ABC transporter, B_12_ transportation	**0.44**	**0.24**	**2.07**	1.1

^*a*^ Values are the fold changes of dNSAF values of the proteins. The bolded values are significantly different according to a *t* test (*P* < 0.05).

^*b*^ H: high (42°C) temperature stress; N: normal (25°C) temperature.

### Co-cultivation and heat stress modulate *Chlamydomonas* and *Sinorhizobium* central metabolism

**Fig 7:**
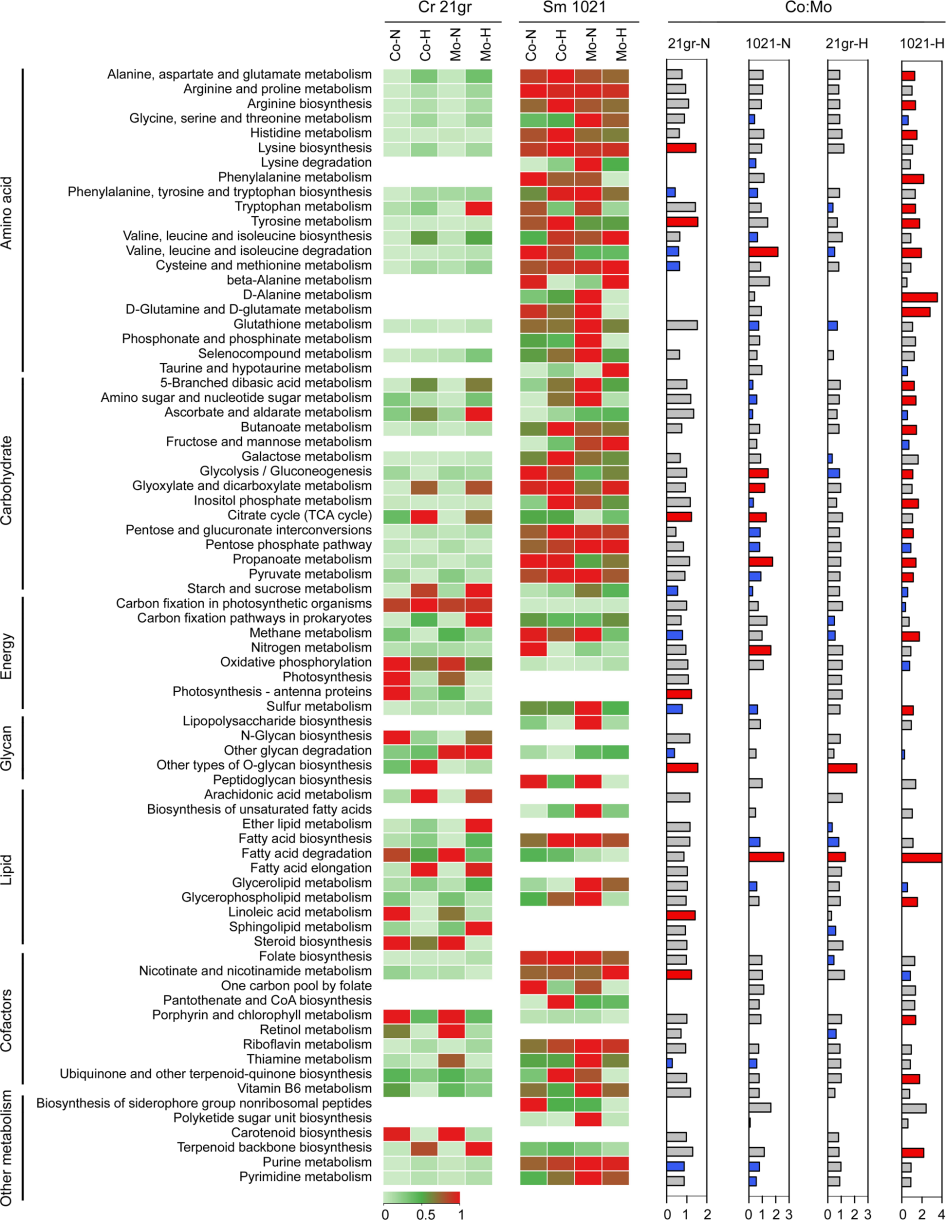
Comparison of metabolism-associated differentially expressed KEGG categories of the *C. reinhardtii* (Cr 21gr) and *S. meliloti* (Sm 1021) proteomes. The heatmaps show the zero-to-one row-scaled (using TBtools) expression of KEGG metabolic categories, and the bars show ratios of each KEGG category in co-cultures vs mono-cultures (Co:Mo) at normal (25°C; 21gr-N and 1021-N) or high (42°C; 21gr-H and 1021-H) temperatures. Bars in red and blue indicate significantly upregulated (ratio > 1.0, *P* < 0.05, *t* test) and downregulated categories (ratio < 1.0, *P* < 0.05, *t* test), respectively, and the others are in gray.

To evaluate how environmental temperature and co-culturing may influence the other metabolic pathways, we analyzed the changes in the expression of central metabolic pathways of *Chlamydomonas* and *Sinorhizobium* based on KEGG (Kyoto Encyclopedia of Genes and Genomes) categories. The results revealed that there are overall 12 pathways in *Chlamydomonas* at high temperature (Fig. 7), for example, tryptophan metabolism [Fold change (FC) = 0.39, *P* < 0.001] and glycolysis/gluconeogenesis (FC = 0.87, *P* < 0.001), which were significantly downregulated, while only fatty acid degradation (FC = 2.59, *P* < 0.001) and other types of O-glycan biosynthesis (FC = 2.17, *P* = 0.019) were upregulated (Fig. 7, blue and red bars under “21gr-H”). In contrast, *Chlamydomonas* co-culture at normal temperature (Fig. 7, bars under “21gr-N”) exhibited increased expression of a variety of amino acid (e.g., lysine, FC = 1.46, *P* = 0.011), citrate (TCA) cycle (FC = 1.24, *P* < 0.001), and co-factor (e.g., nicotinate, FC = 1.24, *P* = 0.04) biosynthesis pathways. However, in *Sinorhizobium* co-cultures at high temperature, the number of upregulated pathways was greater than in co-cultures at normal temperature (24 vs 7), primarily pathways associated with amino acid, carbohydrate, and energy functions (Fig. 7, bars under “1021-H” and “1021-N”). Increased expression of various amino acid metabolism, carbohydrate, and fatty acid degradation likely reflects access to carbon and energy resources derived from *Chlamydomonas* (actively or passively produced) that as a consequence alters *Sinorhizobium*’s metabolic networks. The altered *Sinorhizobium* metabolic network is also reflected in the enrichment of several putative adenylate/guanylate cyclases (e.g., CyaF1 is enriched >11-fold; Table S8) that produce cAMP/cGMP (cyclic adenosine monophosphate/cyclic guanosine monophosphate) which are involved in catabolite control/regulation.

Interestingly, we detected several pathways whose expression was opposing in *Chlamydomonas* compared to *Sinorhizobium* in high-temperature co-cultures. For example, tryptophan metabolism, valine, leucine, and isoleucine degradation, glycolysis/gluconeogenesis, and methane metabolism were upregulated in *Sinorhizobium* but downregulated in *Chlamydomonas* (Fig. 7). However, we did not observe a similar opposing expression pattern in the *Chlamydomonas* and *Sinorhizobium* co-cultures grown at normal temperature, except for valine, leucine, and isoleucine degradation. Exchanges of metabolites between *Sinorhizobium* and *Chlamydomonas* could be facilitated with increased expression of specific transporters during mutualistic interactions, based on different expression levels in the co-cultures compared to mono-cultures (Table 1).

### Co-cultivation and heat stress modulate regulatory networks

Co-cultivation and heat stress alter the abundance of various regulatory proteins, many of which are components of two or one-component systems, or transcriptional activators/regulators (Table 2; Tables S1 to S8). In *Chlamydomonas*, the abundance of several well-characterized signal transduction systems (e.g., MAPK6 and RAPTOR) is among the most highly upregulated proteins in mono-culture, but their expression is greatly modulated by *Sinorhizobium* (Table 2). Interestingly, the downregulation of glycogen synthase kinase 3 (GSK3) (27) was detected in *Chlamydomonas* co-cultures at both normal and high temperatures, suggesting a complex regulation between growth and stress response in the co-cultures.

**TABLE 2:** Expression of representative proteins with signal transduction functions^*a*^

			Fold change			
Protein ID	Gene	Functional description	H:N^*b*^		Co-:Mono-culture	
			Mono-culture	Co-culture	N	H
*C. reinhardtii* 21gr						
Cre07.g327350.t1.2	*CDPK8*	CaMKII_AD domain-containing protein	**0.58**	**1.53**	0.94	**2.49**
Cre08.g371957.t1.1	*RAPTOR*	Regulatory-associated protein of TOR	**8.56**	**2.24**	**3.54**	0.93
Cre12.g499500.t1.1	*SAC3*	Regulator of sulfur deficiency responses	**3.97**	2.17	**1.55**	0.85
Cre12.g508900.t1.2	*MAPK6*	Mitogen-activated protein kinase	**15.47**	3.88	**1.55**	0.39
Cre12.g511850.t1.2	*GSK3*	Glycogen Synthase Kinase 3	0.88	0.77	**0.39**	**0.34**
*S. meliloti* 1021						
Q52990	*phoB*	Phosphate regulon transcriptional regulatory protein	**0.13**	**2.65**	0.8	**16.38**
Q92SA5	*phoR*	Phosphate regulon sensor kinase	**0.14**	2.21	**0.29**	**4.69**
Q92T27	*glk*	Glucose kinase	**1.55**	3.82	**0.15**	**0.37**
Q92Q87	*ntrX*	Nitrogen regulation response regulator	**0.07**	**4.37**	**0.28**	**16.62**
P10577	*ntrC*	Nitrogen regulation response regulator	**0.15**	**2.51**	0.49	**8.3**
P10958	*fixJ*	Regulator of the oxygen limitation response and late symbiotic functions	0.54	0.93	2.49	**4.28**
Q92PM2	*cspA5*	Putative cold shock transcription regulator	**0.22**	**3.18**	**0.34**	**4.88**
Q92NS5	*rpoE1*	Probable RNA polymerase sigma factor	**0.17**	**3.01**	**0.44**	**7.93**
Q92U31	*rpoE5*	RNA polymerase sigma factor	**0.14**	1.76	**0.3**	**3.72**
Q92W49	*sm_b20515*	Blue-light-activated histidine kinase	**0.05**	0.62	0.26	**3.32**
P0C5S3	*actR*	Acid tolerance response regulator	1.29	1.05	**2.34**	**1.89**

^*a*^ Fold change of dNSAF values of the proteins. The bolded values are significant changed accord to *t* test (*P* < 0.05).

^*b*^ H: high (42°C) temperature stress; N: normal (25°C) temperature.

In *Sinorhizobium*, specific regulators are also required to address nutritional and physicochemical conditions during the mutualism. For example, the expression of the phosphate regulon transcriptional regulatory proteins PhoR and PhoB is highly increased in co-culture, particularly under high temperature (Table 2); this is consistent with increased expression of various phosphate transport system components, including PhoD and PhoU (Table S8) and PstB (Table 1). A reduction of Glk (Glucokinase) in *Sinorhizobium* co-cultures may facilitate the utilization of various carbon sources provided by *Chlamydomonas*. Interestingly, several regulatory proteins involved in nitrogen metabolism or oxygen sensing (e.g., FixJ, NtrC, and NtrX) or activated by blue light (Q92W49) were significantly upregulated in *Sinorhizobium* co-cultures exposed to high temperatures.

### *Chlamydomonas* chloroplast responses to heat stress during co-cultivation

A putative mitochondrial thioredoxin o protein (Cre05.g248500.t1.2, FC = 1.55, *P* = 0.003) is upregulated at normal temperature, while the chloroplastic thioredoxin f2 protein (Cre05.g243050.t1.2, FC = 2.82, *P* = 0.012) is upregulated at high temperature (Tables S3 and S4). This suggests that the chloroplast may exhibit greater oxidative stress at higher temperatures than the mitochondria. Additionally, the upregulation of low CO_2_ inducible proteins (e.g., LCI9, FC = 7.24, *P* = 0.035; LCI2, FC = 8.64, *P* = 0.006) and a light stress-induced light-harvesting chlorophyll a/b-binding family protein, OHP2 [FC = 2.61, *P* = 0.009 (28, 29); Tables S2 and S4], suggests that during co-cultivation at high temperatures CO_2_-concentrating mechanism and photoprotection-related pathways are damaged, thereby necessitating repair.

### *C. reinhardtii* co-cultivation induces *S. meliloti* stress responses

Under normal temperatures relative to mono-culture, co-cultivation with *Chlamydomonas* leads to an enrichment of a variety of *Sinorhizobium* proteins (e.g., DegP4 protease, FC = 13.07, *P* < 0.001; heat shock protein HspC2, FC = 9.56, *P* = 0.001; Catalase A KatA, FC = 2.52, *P* = 0.014) implicated in bacterial stress responses and, notably, several distinct universal stress (Usp) domain-containing proteins enriched from 2- to 19.98-fold (Table S7); these proteins are also highly enriched in co-culture at high temperatures but not in mono-cultures (Table S8). Interestingly, several regulators involved in exopolysaccharide/biofilm formation are either enriched (e.g., MucR, FC = 2.82, *P* = 0.002; PecS, FC = 2.51, *P* = 0.009; PtsN, FC = 2.1, *P* = 0.002) or depleted (CbrA, FC = 0.25, *P* = 0.014) (30–33). Moreover, *Sinorhizobium* co-cultures at high temperature are enriched in proteins involved in cyclic glucan and polysaccharide synthesis and export (e.g., NdvB, FC = 5.38, *P* = 0.009; ExoB, FC = 4.89, *P* < 0.01; RkpU, FC = 3.27, *P* = 0.01) as well as in glycogen synthesis and modification (e.g., GlgA1, FC = 2.24, *P* = 0.012; GlgX2, FC = 2.4, *P* = 0.001; GlgP, FC = 2.05, *P* = 0.001; Table S6), suggesting that the environment is carbon rich.

In co-cultures relative to mono-cultures at high temperature, *Sinorhizobium* appears to experience extensive stress based on the enrichment of regulatory (e.g., RpoE1, FC = 7.93, *P* = 0.001; RpoE5, FC = 3.72, *P* = 0.026; CspA5, FC = 4.88, *P* = 0.018; HrcA, FC = 9.97, *P* < 0.001) and heat-shock proteins implicated in general, heat, and envelope stress responses (Table 2; Table S8). Several enriched proteins (e.g., Smc00914, putative ferredoxin reductase, FC = 4.2, *P* = 0.002; GlpD, glycerol-3-phosphate dehydrogenase, FC = 4.1, *P* = 0.048; Table S8), which are involved in environmental stress responses and lipid metabolism, suggests that lipid metabolism may play a role for the stress tolerance of *Sinorhizobium* in the phycosphere. Moreover, proteins involved in facilitating transcription repair (Mfd) and translation (e.g., PrfB, FC = 5.69, *P* = 0.009; RhlE1, FC = 5.25, *P* = 0.001; Rph, FC = 3.12, *P* = 0.014; PpiA, FC = 4.82, *P* < 0.001) or slowing translation (EttA, FC = 8.27, *P* = 0.001) are highly enriched (Table S8). DNA damage also appears to be occurring given the enrichment of a large number of proteins involved in recombination repair (e.g., RecA, FC = 4.56, *P* = 0.002; RecR, FC = 2.05, *P* = 0.02; RecN, FC = 6.03, *P* < 0.001) and DNA replication (DnaX, FC = 3.69, *P* = 0.01; PolA, FC = 3.97, *P* < 0.01; Table S8). Several efflux pumps (e.g., Smc03168, FC = 7.64, *P* = 0.012; Sm_b20346, FC = 7.33, *P* = 0.001; Table 1; Table S8) are highly enriched in co-cultures at high temperature compared to other treatments, suggesting that heat stress leads to more potentially toxic metabolites being produced.

## DISCUSSION

**Fig 8:**
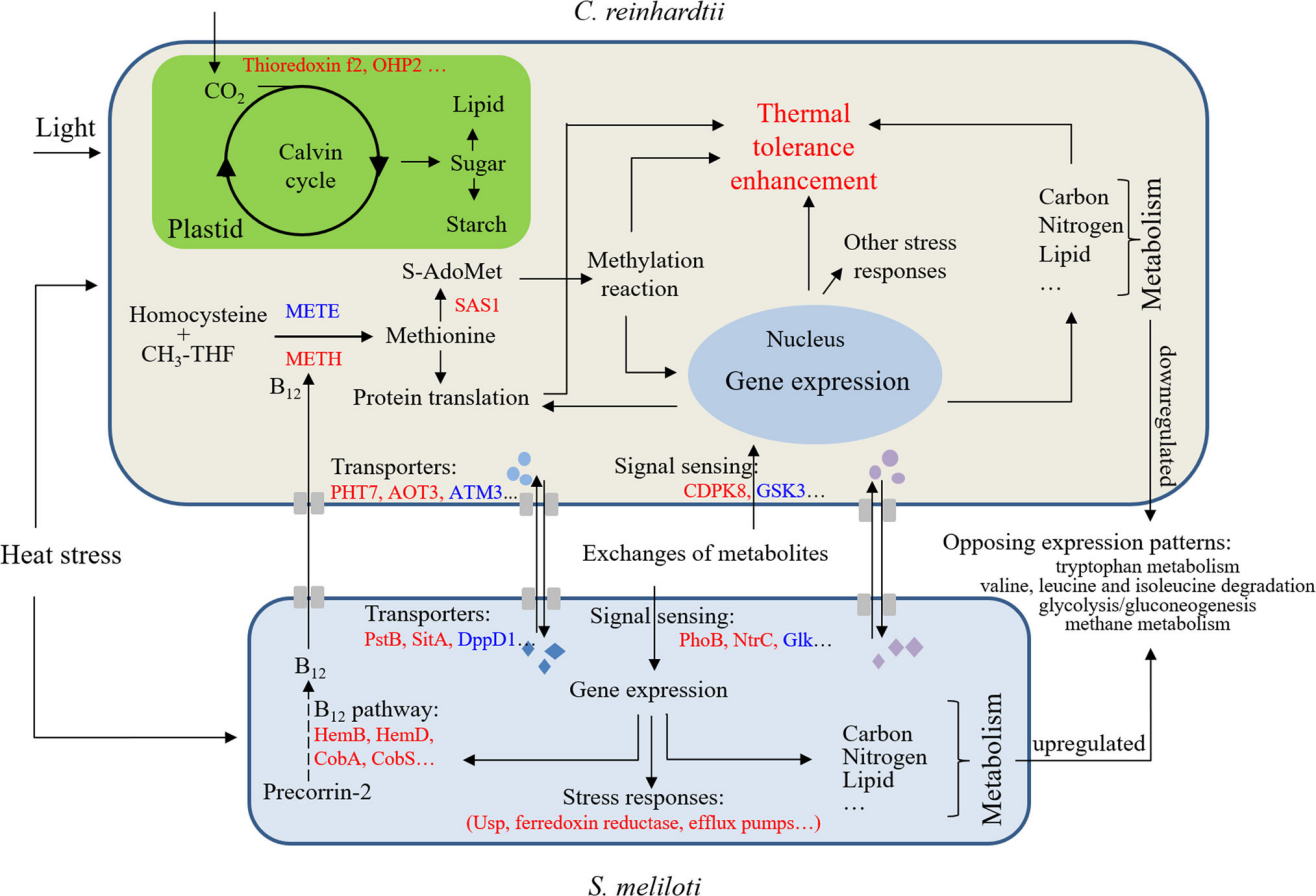
Schematic diagram showing the predicted molecular interactions between *C. reinhardtii* and *S. meliloti* in the coculture vs monoculture during heat stress conditions. Proteins or pathways that are found in higher amounts or are upregulated in cocultures compared with monoculture following a temperature upshift are represented in red, and those that are less abundant or downregulated are blue. The diamond and circular shapes represent the possible exchanged metabolites from *C. reinhardtii* and *S. meliloti*, respectively.

Our findings confirm and expand on the biology of mutualistic interactions between algae and bacteria, highlighting the role of vitamin B_12_ in these interactions (11, 12, 34) and in algal thermotolerance (22, 35). *Chlamydomonas* METE repression occurs either when co-cultivated with *Sinorhizobium* or during temperature upshifts. Since methionine is a direct precursor for S-adenosylmethionine, the major cellular methyl donor, methionine may influence downstream methylation reactions (36, 37). As reported previously, when exposed to high temperature, increased methylations of DNA [such as 5-methylcytosine (5mC) and N^6^-methyladenine (6mA)] and histone may occur, which triggers expression of heat-responsive genes critical for plant basal and acquired thermal tolerance (23, 38, 39). Here, the upregulation of S-adenosylmethionine synthase (SAS1) and certain methyltransferases by heat stress suggests the possible role of B_12_-mediated methionine biosynthesis for supplying sufficient methyl groups for downstream methylation reactions required for the heat stress response. In addition, since methionine is a structural amino acid and the first amino acid in the initiation of most proteins, its availability can be critical for overall protein synthesis and hence cellular physiology. Collectively, this suggests that methylation reactions and protein translation are central factors involved in *C. reinhardtii* thermal tolerance when co-existing with B_12_-producing bacteria (Fig. 8). It also provides a foundation on which to develop new hypotheses for testing how organisms can adapt to a changing environment, and how the environment modulates microbial interspecies interactions (40).

As a key vitamin, B_12_ can only be synthesized by certain prokaryotic organisms, and its direct or indirect release into the environment can be a key metabolite that facilitates recruitment of B_12_ producers to the alga phycosphere (14, 17, 41). Consistent with prior observations (22), we observed upregulation of the B_12_ biosynthesis pathway in *S. meliloti* in co-cultures, particularly under high-temperature treatments (Fig. 5). Several proteins, such as HemB, HemD, and CobA, are enriched in the co-cultures under both normal and high temperatures (Fig. 5), which are consistent with the hypothesis that B_12_ production is regulated during algal-bacterial interactions (42). Why there is increased B_12_ expression is still unclear, whether it is a consequence of a specific signal produced by *Chlamydomonas* that up-regulates *Sinorhizobium* B_12_ expression and/or as a consequence of the nutrients provided by *Chlamydomonas* that alters expression levels. It is important to emphasize that the growth of *S. meliloti* in co-culture relies on carbon released (actively or passively) by *Chlamydomonas* since no other carbon source is provided in the medium. Whether increased expression of the cobalamin biosynthesis pathway is solely intended to meet *Sinorhizobium*’s needs or a direct consequence of stimulation by *Chlamydomonas* for overproduction, the net result is cross-feeding.

In addition to B_12_, other metabolites may also play roles in the thermal tolerance of this *Chlamydomonas-Sinorhizobium* co-culture. Our data showed several opposing expression patterns of metabolic pathways present in high but not normal temperatures of co-cultures (Fig. 7). These results imply that extensive interactions may be occurring based on a division of labor, whereby *Chlamydomonas* provides photosynthate as a *Sinorhizobium* carbon source, and *Sinorhizobium* provides specific energy-expensive metabolites in addition to vitamin B_12_. Of particular interest are differences in tryptophan metabolism since it is considered the most energetically expensive amino acid to produce (43) and one precursor of indole-3-acetic acid (IAA), a key signaling molecule in algal-bacterial interactions (44). It is conceivable that the elevated tryptophan metabolism in *Sinorhizobium* may increase the production of IAA, which can promote the growth of *Chlamydomonas* under high temperature, in a manner that may be similar to the role of tryptophan and IAA in stimulating diatom *Pseudo-nitzschia multiseries* cell division when co-cultured with bacterium *Sulfitobacter* (44).

Another important question is how B_12_ and the other metabolites are transported and sensed. In bacteria, exogenous corrinoids are transported into the cell via an ABC transport system composed of Ton-B-dependent outermembrane permease, a periplasmic-binding protein, a cytoplasmic membrane permease, and an ATPase (BtuB, F, C, and D) (45). The increased expression of BtuC and BtuF in *Sinorhizobium* co-cultures (Table 1) suggests that during co-cultivation, leakage of cobalamin out of the cell is potentially compensated for by re-importing cobalamin or cobinamide back into *Sinorhizobium* for salvaging purposes (8, 46, 47). However, whether B_12_ secretion is passive or, if active, how it is regulated in co-cultures still remains unclear. In diatoms *Phaeodactylum tricornutum* and *Thalassiosira pseudonana*, cobalamin acquisition protein 1 (CBA1) has been implicated in cobalamin uptake (48). Recently, CBA1 homolog protein (Cre02.g081050.t1.2) was identified in *Chlamydomonas* (49). Our data showed that CBA1 expression is slightly, albeit not significantly, increased in the mono-cultures after a temperature upshift (FC of Mo-H:Mo-N = 1.08, *P* = 0.53) and the co-cultures under normal temperature (FC of Co-N:Mo-N = 1.077, *P* = 0.69). However, its expression is decreased slightly in the co-cultures when comparing high vs normal temperature (FC of Co-H:Co-N = 0.619, *P* = 0.086) or when comparing co-cultures vs mono-cultures under high temperature (FC of Co-H:Mo-H = 0.617, *P* = 0.12). These patterns imply that the *Chlamydomonas-Sinorhizobium* microenvironment can be rich in B_12_ under high temperature since CBA1 expression can be downregulated when B_12_ is abundant as reported recently (49), or *Chlamydomonas* does not solely rely on CBA1 to obtain B_12_ from *Sinorhizobium* during heat stress. Additionally, the other DEPs associated with signal transduction and transportation (Tables 1 and 2) imply that there are comprehensive interspecies recognitions and other metabolic communications within this coculture.

Prolonged heat stress causes enhanced rates of protein unfolding as reflected by increased expression of various molecular chaperones and proteases designed to correct folding and prevent protein aggregation (50, 51). In *Chlamydomonas*, heat stress increased their abundance dramatically, but co-cultivation with *Sinorhizobium* had no apparent ameliorative effect. Interestingly, in co-cultures at normal temperatures, there was a small (~1.62-fold) increase in the expression of a variety of heat shock proteins (e.g., peptidylprolyl isomerases, HSP-like chaperone superfamily proteins; Table S3). Similarly, in co-cultures at normal temperature (Table S3), there was a slight increase in expression by *Chlamydomonas* of proteins for glutathione S-transferase, thioredoxins, and a manganese superoxide dismutase involved in the oxidative stress response. Collectively, these findings indicate that *Chlamydomonas* interactions with *Sinorhizobium* are modestly stressful, which has also been observed in other alga-bacterial interactions (16), although why is currently unclear. Protective strategies, such as expression of chaperone or universal stress proteins, to ameliorate the stress or to increase expression of proteins for exopolysaccharide/biofilm formation to minimize the effects of stress indicate that there is a fine balance between beneficial and detrimental consequences of the interaction.

Compared with their mono-cultures, the regulation of growth and stress responses by *Chlamydomonas* and *Sinorhizobium* in co-culture is complex. This is reflected in changes in the expression of some key, multi-function regulators. For example, glycogen synthase kinase 3 (GSK3) proteins, the highly conserved serine/threonine kinases in eukaryotes, are required for growth, development, and biotic and abiotic stress responses in plants (27). In *Chlamydomonas-Sinorhizobium* co-cultures, GSK3 expression levels are downregulated (Table 2). This suggests that one or more *Sinorhizobium*-derived signals can greatly reduce the expression of GSK3 independent of environmental temperature. This reduction may result in either the common (e.g., the shared DEPs in Fig. 2C) or specific downstream responses (e.g., the unique DEPs in Fig. 2C) by *Chlamydomonas* in the presence of *Sinorhizobium*. Future functional studies of these proteins will contribute to our understanding of the molecular mechanism of algal-bacterial interactions.

The mutualistic exchange of nutrients between algae or diatoms and microbes (in particular, algae and B_12_-producing bacteria) has become an area of increasing interest given the impact that these interactions could have on global nutrient cycling (12, 16, 22, 44). Thus far, a common molecular interaction mechanism is still not well known, suggesting that there is a certain degree of specificity in the interaction, likely in a context-dependent manner. Here, our label-free quantitative proteomics approach has provided new insights into the molecular basis of algal-bacterial interactions and highlights the balance between the beneficial properties required for maintaining co-existence (metabolite exchange) and the potentially detrimental challenges each partner must overcome to survive. This information provides additional insight into the processes governing community interactions and function in the phycosphere.

## MATERIALS AND METHODS

### Growth of *Chlamydomonas* and *Sinorhizobium*

*C. reinhardtii* 21gr (CC1690, Chlamydomonas Resource Center) and *S. meliloti* 1021 (52) were maintained on Tris-acetone-phosphate and Tryptone-Yeast Extract media, respectively (53, 54). Co-inoculation of *Chlamydomonas* and *Sinorhizobium* and thermal tolerance assays were performed as described previously (22), with the exception that the thermal tolerance assays were performed in liquid cultures rather than on solid media. Briefly in the thermal tolerance assays, *Chlamydomonas* and *Sinorhizobium* were inoculated in minimal salts (MM) liquid medium (55) at approximately a 1:10 (*Chlamydomonas: Sinorhizobium*) cell density ratio. For the *Sinorhizobium* mono-culture controls in MM medium, 0.1% sucrose was supplemented as the carbon source. These cultures were grown under continuous illumination (120 µmol photons m^−2^ s^−1^) at 25°C. For inducing a temperature upshift, mono- and co-cultures were grown at 25°C for 3 days prior to transferring cultures to 42°C.

### Protein extraction and digestion

Two days after the temperature upshift, the mono- and co-cultures of *Chlamydomonas* and *Sinorhizobium* were collected, and protein samples were prepared as described previously (56). Briefly, cells were collected and resuspended in a lysis buffer consisting of 20 mM Tris-HCl (pH 7.5), 150 mM NaCl, 0.1% DDM (*N*-dodecyl-β-d-maltoside), 50 mM DTT (dithiothreitol), and 1 × Roche protease inhibitor cocktail. Cells were lysed with a sonication probe for 10 min on ice (135 W output, 5 s on, and 5 s off), and the whole cell lysate was centrifuged (12,000 g at 4°C for 15 min) to remove cell debris. The supernatant-containing proteins were precipitated with −20°C cold acetone for 2 h, and the precipitate was dissolved in 50 mM ammonium bicarbonate digestion buffer. Protein concentration was determined using the BCA Protein Assay Kit (Beyotime, Beijing, China). Protein aliquots (100 µg) were reduced with 10 mM dithiothreitol for 0.5 h at 37°C before they were alkylated by 15 mM iodoacetamide in the dark for 30 min. Then, the proteins were digested with trypsin (Promega, UK) at a ratio of 1:45 (wt/wt) for 18 h. Desalting was performed using ZipTip C18 columns solid phase extraction (Millipore) and 0.1% formic acid (FA). The peptides were then eluted with 75% acetonitrile and 0.1% FA.

### LC-MS/MS analysis

Proteomics samples were analyzed using an Exactive Plus Orbitrap Mass Spectrometer coupled with an EASY-nLC 1200 System (Thermo Fisher Scientific, Rockford, USA). Briefly, about 1 µg of protein sample was separated using a C18 column (150 Å pores, 15 cm length, and 100 µm diameter) at a flow rate of 300 nL/min for 120 min. The segmented linear gradient of mobile phase A (5% acetonitrile/0.1% FA) and 10%–80% of mobile phase B (90% acetonitrile/0.1% FA) was used to elute peptides prior to nanoelectrospray ionization at 2.0 kV. Mass spectrometry was performed using a data-dependent acquisition mode, and full precursor scans were collected with a mass range ranging from 350 to 2,000 m/z at 70 k resolution. The 20 most abundant precursor ions per scan were chosen for higher-energy collisional dissociation fragmentation. For MS-MS analysis, a resolution of 17,500 and an isolation window of 1.8 m/z were used in the Orbitrap Mass Spectrometer. The dynamic exclusion duration was 40 s, and the automatic gain control (AGC) target value was 5e^4^.

‍Protein sequences of *Chlamydomonas* and *Sinorhizobium* were searched using Proteome Discoverer 2.1 against *Chlamydomonas* protein database (v5.6, www.phytozome.net) appended with NCBI chloroplast and mitochondrial databases (BK000554.2 and NC_001638.1) and *S. meliloti* Uniprot proteome database, respectively. Only proteins detected in at least three replicate samples of treatment and included at least one unique peptide [false discovery rate (FDR ≤ 0.01)] met our criterion to be considered as a measurable protein.

### Data analysis

For protein expression level comparisons, dNSAF was calculated for each protein as previously described (24). The data of each treatment were derived from four independent replications, except for *Chlamydomonas* mono-cultures which were from three independent replications. Null spectral count values were replaced with a small spectral fraction before performing the calculations (57). Natural log-transformed dNSAF data were compared using Student’s *t* test for two groups or one-way ANOVA followed by Duncan post-hoc tests for multiple groups with SPSS 19 software (IBM, USA). DEPs with FC ≥ ±1.5 and *P* < 0.05 were considered as upregulated and downregulated proteins, respectively. GO enrichment analyses of DEPs were performed using TBtools (58). Pathway analyses were performed according to KEGG category annotations (WWW.KEGG.JP) and the approaches described previously (24).

## Data Availability

Mass spectrometry proteomics data have been deposited in the ProteomeXchange Consortium database (http://proteomecentral.proteomexchange.org) via the iProX partner repository (59) with the data set identifier IPX0004233001.

## SUPPLEMENTAL MATERIAL

Supplemental material spectrum.00219-24-s0001.xlsx Tables S1 to S9.

ASM does not own the copyrights to Supplemental Material that may be linked to, or accessed through, an article. The authors have granted ASM a non-exclusive, world-wide license to publish the Supplemental Material files. Please contact the corresponding author directly for reuse.
